# Empirical analysis based on the related factors of college students’ mental health problems

**DOI:** 10.3389/fpsyg.2022.997910

**Published:** 2022-09-26

**Authors:** Huang Zhenhua, Wang Nan

**Affiliations:** ^1^College of Materials Science and Engineering, Qiqihar University, Qiqihar, Heilongjiang, China; ^2^College of Foreign Languages, Qiqihar University, Qiqihar, Heilongjiang, China

**Keywords:** college students, mental health, optimism, relevance, interpersonal relationships

## Abstract

Interpersonal relationship is one of the important factors affecting college students’ mental health. The relationship between interpersonal relationship and college students’ mental health has also become a large number of academic research topics. In order to explore whether there is a correlation between optimism and college students’ mental health, and if so, what kind of situation it presents. Based on literature review, mathematical statistics and questionnaire survey, this study optimized the iterative process of clustering algorithm. Extract valuable parts from a large amount of precipitation of students’ psychological data, establish data models, and provide decision-making guidance for managers. The results show that there are significant differences between optimists and pessimists in optimistic factors and pessimistic factors. Optimists score significantly higher on optimistic factors than pessimists, while pessimists score significantly lower than pessimists. Conclusion optimism can significantly alleviate life stress and intervene psychological crisis.

## Introduction

With the continuous progress of society, the lifestyle of college students is affected by many modern factors. With the increase of modern elements in life style, the objective health status and healthy values of college students are constantly changing. The mental health of college students has attracted the attention of all walks of life, and there are more and more researches on CSMH (college students’ mental health). CSMH is a multi-dimensional organic system. The pressures and confusions faced by college students, such as social competition, employment situation, life adaptation, interpersonal communication, etc., all have a great impact on their mental health and growth, and their mental health has become the focus of social attention (Shervin and Maryam, 2018). Therefore, colleges and universities should pay attention to the education of secondary vocational students. Correctly understand and accurately position the problems of secondary vocational school students. Actively consider the feasible ways to innovate the teaching of secondary vocational students under the new situation, and constantly promote the comprehensive development of secondary vocational students.

The mental health problems of college students have been concerned by parents, teachers and mental health workers. According to relevant statistics, among college students, “the number of retired students caused by psychological disorders or psychosis accounts for about 30% of the total number of retired students and is on the rise.” The survey results of the Institute of mental health of Peking University on 16 universities in Beijing show that the proportion of college students who quit or quit school due to psychological disorders in the total number of students who quit or quit school has an increasing trend year by year. The number of students who quit or quit school due to psychological disorders or mental diseases in some universities accounts for about 50% of the number of students who quit or quit school. Research shows that the main problems of medical students are emotional disorders, mainly manifested in depression and anxiety. Although the government and professionals continue to call for reducing the burden on students and carrying out quality education, medical students have an increasing trend in the incidence of psychological disorders due to the rigorous requirements of disciplines on knowledge, the increase in the number of medical students and the upcoming employment pressure. In particular, nursing students, as the main body of the future health care technology talent team, not only need to cultivate superb nursing skills, but also need to have good psychological quality. However, due to the boring theoretical and professional knowledge of nursing major and less communication with other colleges, most of them are girls, which is relatively lack of humanistic atmosphere. Some studies have pointed out that the decrease of BDNF in serum leads to depression. Studies have confirmed that the pathogenesis of depression is not only related to biological factors, but also related to social and psychological factors.

Quality education is to make the educated develop harmoniously not only in physical quality, but also in psychological quality and social and cultural quality. Develop in an all-round way in morality, intelligence and physique. For a long time, many people pay more attention to physical health, but less attention to mental health. Adolescence is the transition period from immaturity to maturity. Individuals in this period. While the physiological level is significantly improved, its psychological development characteristics, especially in intellectual development, emotional and will performance, personality and language performance, have unique development characteristics. In the context of the new curriculum reform, the students we cultivate should not only have rich cultural knowledge, strong physique, but also have healthy psychological quality. Physical exercise can not only strengthen the body, but also promote people’s mental health, eliminate people’s negative emotions (including depression, depression, sadness, fatigue, depression, etc.), cultivate people’s positive emotions, meet people’s psychological needs, and keep people mentally happy. At present, college students’ physical exercise and health have received the attention of many modern colleges and universities. This chapter will mainly analyze and explain college students’ physical exercise and mental health, the nutritional supplement of College Students’ physical exercise, and the health care of College Students’ physical exercise. Exercise gives participants strong emotional feelings, realizes participants’ Self-worth from another angle, and makes people obtain spiritual pleasure. Physical exercise can delight people’s spirit and maintain a healthy body, which is of great significance to the improvement of learning efficiency, life and quality of life.

The innovative contribution of this study is to optimize the iterative process of clustering algorithm based on mathematical statistics and questionnaire survey. Extract valuable parts from a large amount of student psychological data, establish data models, and provide decision guidance for managers. The results show that there are significant differences between optimists and pessimists in optimistic and pessimistic factors. Optimists score significantly higher on optimistic factors than pessimists, while pessimists score significantly lower than pessimists. Conclusion optimism can significantly relieve life pressure and intervene psychological crisis. Stress is an important cause of College Students’ psychological crisis. The greater the pressure, the greater the impact on students’ mental health. Gender, grade and major were positively correlated with mental health scale, social well-being and positive function, while self-centered network density was negatively correlated with mental health continuum scale and mental well-being subscale.

## Related work

From the psychological point of view, domestic universities have just started to study and discuss the education and teaching of college students, which cannot meet the needs of society and adapt to the development of the situation. In order to achieve the scientificity and effectiveness of mental health education, all universities are engaged in active information technology research and development. Tang et al. verified the regulating effect of self-transcendence life meaning on CSMH, and proved that self-transcendence life meaning can be used as a stress coping resource for college students (Tang et al., 2021). Vaughn et al. pointed out that CSMH includes college students’ self-cognition and self-improvement, emotion and emotion regulation, frustration and pressure, personality optimization and will exercise (Vaughn et al., 2016). Ying pointed out that mental health education, as an important part of China’s basic education curriculum system, is an indispensable part of modern school education (Ying, 2021). Wang’s electronic archives theory is applied to the network-based CSMH system, and the database table structure and planning of psychological archives management and storage meet the needs of electronic archives management and retrieval (Wang, 2020). Compared with the short-term emotional effect of sports, there are few studies on the long-term emotional effect of sports at present. Existing studies suggest that the long-term emotional effect may be related to the content of sports. If individuals engage in noncompetitive and pleasant sports, it will promote the long-term emotional effect. In addition, the intensity of sports also has an impact on the long-term emotional effect. For example, regularly engaging in moderate intensity sports is conducive to the improvement of long-term emotion. Zhangqian et al. established a mental health scale for children and adolescents, which consists of several items through the research of hundreds of primary and secondary school students, involving five factors: cognition, thinking and language, emotion, will and behavior, and personality characteristics (Zhang and Chen, 2019). Wei et al. think that teachers’ mental health is correct cognition, strong psychological adjustment ability, multi-dimensional psychological orientation, beautiful emotion and good self-image (Wei et al., 2020). Debate et al.’ s research shows that the victim’s speculation about the aggressor’s aggression motive has a very profound influence on optimism (Debate et al., 2018). Researchers also believe that among a series of social cognitive factors, attribution mode has the most profound significance for optimism (Tang and Xu, 2016; Xue and Liu, 2018). For example, through the research on the memory occurrence of husband and wife, others not only verified the relationship between intimacy and optimism, but also proposed that the cognitive factor of the explanation of events is the intermediary between them.

Under the background that universities pay attention to health education to improve college students’ comprehensive quality, studying the relationship between college students’ physical exercise and mental health is of great significance in both theory and practice for building a modern healthy university and cultivating useful talents based on overall physical and mental health (Taylor, 2019; Fu et al., 2021). This study intends to make an empirical analysis of the relationship between college students’ optimism and mental health, and establish a model of the relationship between college students’ optimism, daily adaptation and psychological distress, so as to provide some references for college students to better face and deal with daily stress problems and improve their physical and mental health. Exploring the relationship between optimism and CSMH aims to explore the realistic problems of college students’ optimism, the realistic problems of CSMH, and the influence of college students’ optimism on their mental health and mental disorders in practice.

## Research method

### Research objects

In this study, 350 college students were selected by cluster random sampling, taking into account factors such as gender, grade, registered permanent residence, whether they have only one daughter, arts and sciences, etc. to conduct a questionnaire survey. 325 valid questionnaires were collected, with an effective rate of 92.9%. Excluding those who answered incompletely and those who answered clearly, 312 questionnaires were kept.

According to the principle of three standard deviations, 302 valid questionnaires were finally obtained. At the end of the questionnaire, the researcher provided the contact information so that those who have doubts can ask at any time.

### Research tool

Symptom Checklist-90 (SCL-90) was used to assess the mental health status in the last week. The clonal Bach coefficient α of 10 factors in this survey is between 0.876 and 0.679.

Mental health scale for college students, which consists of 104 items. The whole scale includes 12 dimensions. The internal consistency reliability of each sub-scale is 0.764 ~ 0.893, which has a high positive correlation with each dimension of SCL-90 (Wang and Shi, 2018; Fruehwirth et al., 2021). In this study, the internal consistency coefficient α coefficient of the scale reached 0.906.

The self-made “Optimism Scale for College Students” refers to the theory of optimistic personality, combines the definition of optimistic personality in this study, and compiles some items by itself. Based on the investigation and interview and life orientation test, it forms a 35-item optimism scale for college students. The higher the score, the less pessimistic it is.

### Research hypothesis

Reference (Xiao et al., 2021) points out that physical exercise can effectively reduce the addiction of smartphone users. The comparison and further evaluation of the long-term effects of different types of exercise intervention in the treatment of PSU remain to be studied. Reference (Wongkoblap et al., 2017) discusses the scope and limitations of cutting-edge technologies used by researchers for mental health prediction and analysis, and reviews related issues in this research field. In the research on the relationship between optimism and mental health, the hypothesis is put forward, and it is the main way of thinking and way to conduct research by establishing models and questionnaires. Personally, the two research orientations are actually not contradictory, temperament optimism is a stable personality trait, optimistic explanation style is a cognitive ability, an attributive explanation of life events, and how to make your own attribution in the face of common life stress events. Optimism is actually rooted in historical materialism. Historical materialism holds that the people are the driving force to create world history. As long as we trust and rely on the people, we can overcome any difficulties and obstacles.

From the perspective of the influence mechanism of college students’ optimism on their mental health, college students’ optimism factors can be regarded as stimulus (or “antecedent factors”), and adaptation and psychological distress symptoms can be regarded as the final response (or “outcome variables”). The greater the optimism of college students, the worse their adaptation, the more serious their psychological distress and the easier it is to cause a series of psychological obstacles such as anxiety and depression. However, in the past, the relevant research tools were often non-local research tools, and the measurement of CSMH status would have great cultural differences. What is more, how different sources of optimism affect college students’ adaptation in different aspects, and how college students’ maladjustment in school life can cause psychological distress, etc., need further in-depth analysis and discussion. A person’s emotions are formed by the meaning he gives to life and the goals he sets for himself. To a large extent, emotion can manipulate the body, but it does not depend on the body. It mainly depends on the individual’s goals and lifestyle. Many people mistakenly believe that the good mood of optimism is destroyed or given by external factors. In fact, people’s best emotions come from their own hearts. Optimistic contact with your emotions is the real source of your best emotions. Different ways of emotion regulation have different predictions for different aspects of social adaptation. Ignoring the regulation and readjustment of negative emotions can well predict the overall social adaptation. Pay attention to regulation through positive emotions, and positive emotions are naturally regulated. It has a certain predictive effect on learning adaptation. Therefore, college students’ emotional regulation is closely related to social adaptation. Different emotions have different effects on the performance of theory of mind: compared with neutral emotions. Positive emotions significantly improved the performance of theory of mind, while negative emotions reduced the performance of theory of mind. Self-esteem is a psychological mechanism for individuals to adapt to social and cultural environment. Empirical research shows that individuals with high self-esteem are more accustomed to using effective emotion regulation strategies such as cognitive reappraisal. Even in the face of negative events, they can more calmly adjust their emotions and avoid the negative impact of external threats on themselves. When individuals produce positive emotions, they are in a comfortable state. Without anxiety and tension, the scope of individual thinking and attention has been expanded. In addition, the expansion of thinking attention behavior will also allow individuals to develop other resources, such as physical resources, intellectual resources, psychological resources and psychological resources. From the perspective of evolutionary psychology, positive emotions help individuals better cope with problems in growth and development. At the same time, positive emotions can also expand the scope of individual thinking.

CSMH can be understood as: students can adapt to the changing campus environment and social environment, keep a good state of cognitive ability, emotional reaction and will action, and have normal learning and life control ability. Generally, the behavior of college students with sound will has a clear purpose, can consciously control their behavior, and can make timely analysis and decisions according to the changes of environmental conditions, and adopt scientific and effective methods to solve various problems encountered. Psychologically healthy college students have a clear and objective cognition of their social environment and its future development trend, and their thoughts and behaviors can adapt to the changing social environment and meet the social requirements to the greatest extent. Their healthy psychology not only has a great influence on the growth of college students themselves, but also has a profound influence on the smooth development of the whole society and even the whole nation.

In China, there are few empirical studies on self-actualization, and there is no empirical study on college students’ self-actualization, and there is no previous research record on college students’ self-actualization and mental health. The research hypothesis of this paper is:

*Hypothesis 1*: The overall level of self-realization needs of college students is low;

*Hypothesis 2*: Optimism is positively correlated with life satisfaction, social support and efficacy; It is negatively correlated with life stress, depression and psychological crisis.

*Hypothesis 3*: Self-esteem and interpersonal accommodation are the mediating variables of optimism affecting depression, happiness index and life satisfaction.

### Method model

The strengthening of college mental health education and the improvement of college students’ psychological quality are not only of great significance to their all-round development in terms of physical and mental health, morality, intelligence, physique and beauty during their studies at school, but also have a far-reaching impact on their personal life-long development and the improvement of the overall quality of the nation’s new generation.

Clustering technology has been widely used in many applications, including pattern recognition, data analysis, image processing and market research. Through clustering, people can identify dense and sparse areas, and thus discover the global distribution pattern and the interesting relationship between data attributes. Therefore, it is of great practical significance to discuss the performance and application scope of different algorithms. This method integrates cluster analysis into CSMH analysis, so that the school can better understand and master all aspects of students’ information.

Using the basic idea of combining statistical methods with data mining algorithms, some existing effective statistical methods are combined with data mining algorithms to produce some efficient statistical methods and increase the efficiency of cluster analysis. Similarity measurement method (Góngora et al., 2018), it is necessary to select a suitable criterion function (Jorge et al., 2016; Qi et al., 2021).

Let the mixed sample set $X=\left\{x_{1},x_{2},\ldots ,x_{n}\right\}$ , based on some correlation, classify the sample aggregation into C separated subsets, each subset $X_{1},X_{2},\ldots ,X_{e}$ is a type, and contains $n_{1},n_{2},\ldots ,n_{c}$ samples, respectively. In order to measure the instruction of clustering algorithm, the error square sum criterion function is adopted, and the definition formula is as follows:


(1) $$J_{C}=\overset{C}{\underset{j=1}{\sum }}\overset{n}{\underset{k=1}{\sum }}x_{k}^{\left(j\right)}-m_{j}{}^{2}$$

(2) $$m=j1/n_{j}\left(\overset{n}{\underset{j=1}{\sum }}xj\right)j=1,2,\ldots ,c$$

In the formula, mj represents the average value of samples in the j th class, and $n_{j}$ represents the number of samples in the j th class. According to the definition of the criterion function in the above formula, it is not difficult to find its value j cluster centers and samples in each cluster.

In the whole process of association rule mining, the time spent in finding all frequent itemsets from transaction sets is the main step, so most of the research on association rule mining algorithms now focuses on reducing the time spent in this process. The association rule can be expressed as X⇒Y , where X⊂I,Y⊂I,X∩Y=∅ . Support S indicates the frequency of rules appearing in transaction sets, and confidence C indicates the frequency of Y appearing in things containing X .

Their mathematical formula is as follows:


(3) $$s\left(X\Rightarrow Y\right)=\frac{\sigma \left(X\cup Y\right)}{M}$$

(4) $$c\left(X\Rightarrow Y\right)=\frac{\sigma \left(X\cup Y\right)}{\sigma \left(X\right)}$$

Where M represents the number of transactions.

Information annoyance is mainly used in information theory to measure the degree of ordering of a system. Information annoyance is inversely proportional to the degree of ordering of a system, and its value gradually decreases with the increase of the degree of ordering of a system. The FCM (Fuzzy C-Means) algorithm classifies data by iterative optimization of objective function.

There are n sample points $x_{1},x_{2},\ldots ,x_{n}$ , which are divided into c class sets. $Y_{j}=\left(x_{i}|x_{j}\in y_{j}\right)$ is used to represent the j class set, and the information entropy of the j class is:


(5) $$s=\overset{c}{\underset{j=1}{\sum }}\underset{x_{i}\in y_{i}}{\sum }p_{ij}\ln p_{ij}$$

The difference $s_{j}-s_{j-1}$ of the information entropy value when the sample jumps from the j state to the j−1 state is called the jumping value of the information entropy. At this time, the number of classes tends to be stable, so the number of classes at this time is the final number of classes.

**Figure 1:**
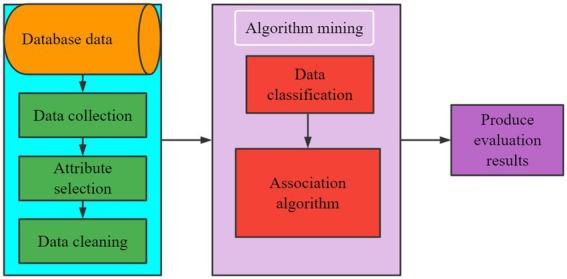
Data mining process of CSMH analysis.

The implementation process of CSMH analysis is to collect college students’ health data at first. After selecting and cleaning the data attributes, the useful data is built into a comprehensive psychological test database. The flow chart of data mining of CSMH analysis is shown in Figure 1.

Through stimulating students’ self-confidence and self-awareness at a higher level, students’ psychological activities are guided to change, and their adaptability to complex environment is gradually strengthened, so that students’ potential can be efficiently explored and brought into play.

People’s mental health is a very complicated dynamic process, and the factors that affect mental health and lead to mental illness are also extremely complicated. After investigation, it is found that the main reason for CSMH problems in recent years is stress. Some college students are influenced by the bad social atmosphere, which breeds the habit of attaching importance to material enjoyment and comparing with each other in life. College students, who are more financially stressed, feel ashamed and belittle themselves, lift their heads in front of classmates, and feel that they are not accepted, so they feel inferior, depressed, annoyed, and even commit suicide or crime.

Mahalanobis distance represents the distance between a point and a distribution. It is an effective method to calculate the similarity of two unknown sample sets. It is not affected by dimension. The Mahalanobis distance between two points is independent of the measurement unit of the original data. The Mahalanobis distance between two points calculated from standardized data and centralized data (that is, the difference between the original data and the mean value) is the same. Mahalanobis distance can also eliminate the interference of correlation between variables. The Mahalanobis distance between two points calculated by standardized data and centralized data (that is, the difference between the original data and the mean) is the same.


(6) $$D\left(X_{i},X_{j}\right)=\left(X_{i}-X_{j}\right)*S^{-1}*\left(X_{i}-X_{j}\right)$$

Where $X_{i},X_{j}$ is the vector composed of m indicators of the i th and j th samples, respectively, and S is the covariance of the sample matrix.

For association rules, many algorithms use the framework of “support-confidence.” Such a structure sometimes produces some wrong results. If we set the support and confidence level low enough, then we will get two contradictory rules. In practical application, subjective and objective metrics should be used at the same time. First, the objective metrics should be used to filter rules, and then the subjective metrics should be used to select interesting rules according to the different needs of users. Generally, when evaluating the quality of rule A⇒B , at least three factors should be considered: coverage, integrity and credibility factor.

So the degree of interest is defined as:


(7) $$\mathrm{Interest}=\left(A\Rightarrow B\right)=\frac{P\left(A|B\right)}{P\left(A\right)P\left(B\right)}$$

**Figure 2:**
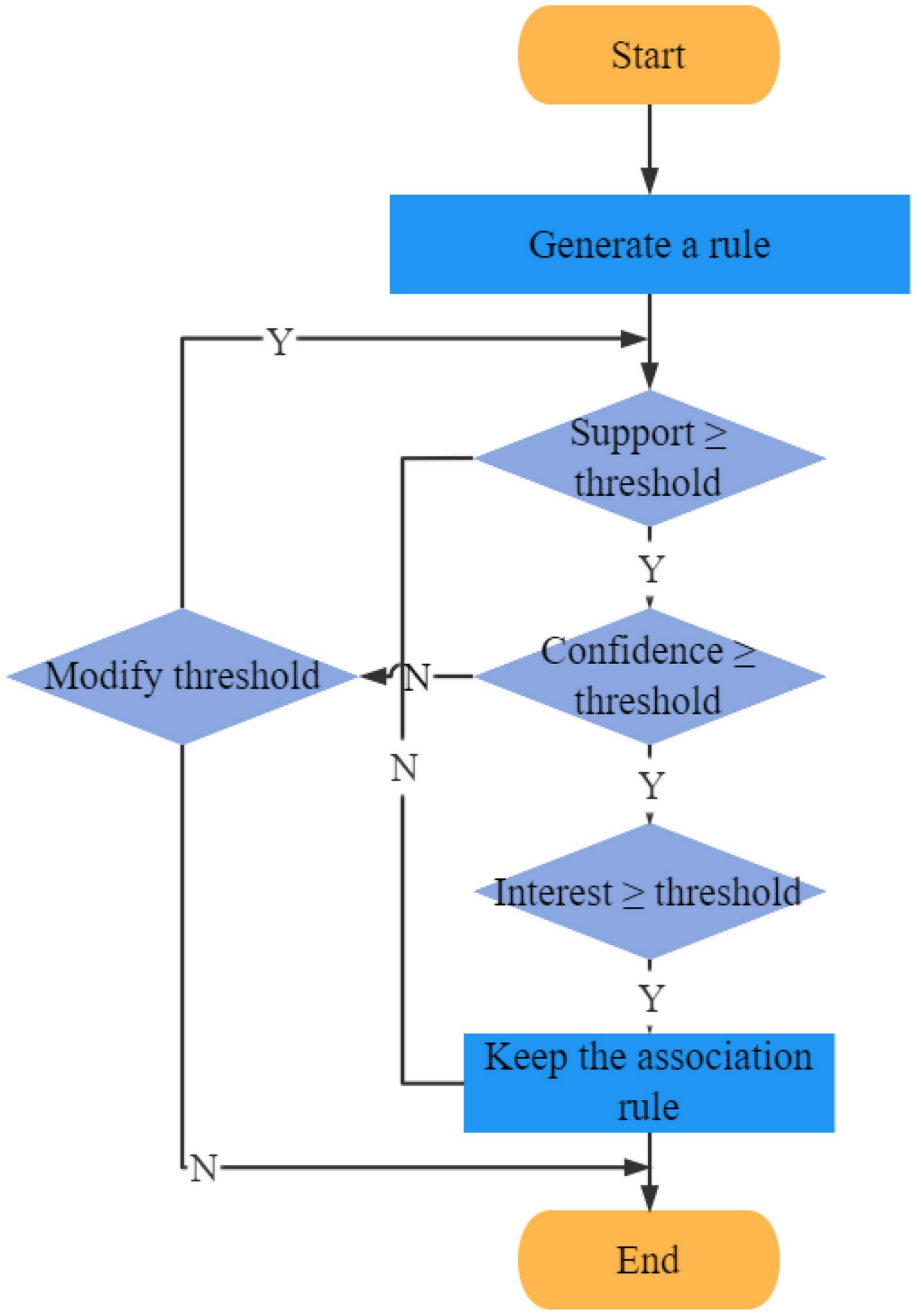
Flow chart of association rule mining.

In this paper, the association rules mining with interest degree is adopted, and its process is as follows: Figure 2.

Based on the grey evaluation of triangular whitening weight function, the psychological state evaluation of college students is constructed, and the geometric midpoint of $\left[a_{1},a_{2}\right],\left[a_{2},a_{3}\right],\left[a_{3},a_{4}\right]$ among three districts in the grey number classification table of psychological health evaluation indexes of college students is determined:


(8) $$\lambda_{k}=\frac{\left(a_{k}+a_{k+1}\right)}{2},k=1,2,3$$

When judging a large concept, there are often many determinants, so these factors are treated according to the same attribute, the same or similar ones are classified into one category, and then the weights are distributed, and then comprehensive evaluation is made. Similarly, the determinants in each category depend on many sub-factors or low-level factors, so the low-level sub-factors are also comprehensively evaluated.

Divide the factor set $U=\left\{u_{1},u_{2},\ldots ,u_{n}\right\}$ into a subset or a unit according to similar characteristics, and divide it into s subsets:


(9) $$U_{i}=\left\{u_{i1},u_{i2},\ldots ,u_{in_{i}}\right\}$$

Among them, i=1,2,…,s .

**Figure 3:**
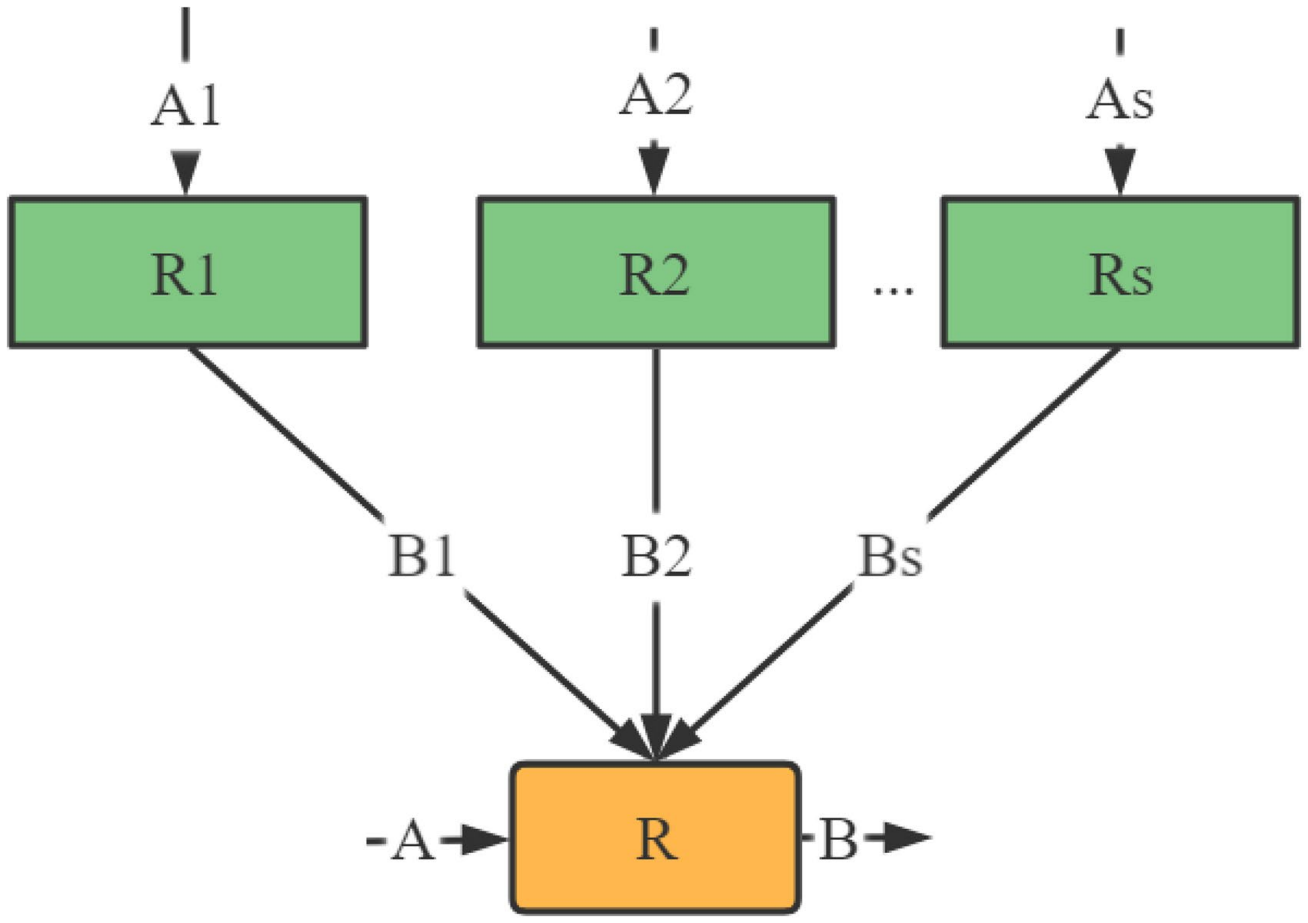
Two-level evaluation model.

When judging, each sub-factor set is regarded as independent, and comprehensive decisions can be made separately. Figure 3 gives an intuitive explanation of the two-level decision model.

If each sub-factor set $U_{i}$ is still huge and contains multiple types and quantities of factors, then we will divide it again, and the result will be a three-level model and so on. Finally, according to the principle of maximum subordination, corresponding to the result set of fuzzy comprehensive evaluation, the results of CSMH are obtained, namely (good, normal, slightly abnormal, seriously abnormal).

## Result analysis

### A study on the differences between optimists and pessimists

In this survey, we selected the Department of management engineering and the Department of mechanical and electrical engineering of our department to conduct the survey. The ratio of male to female students is 1:1. The method of sampling survey is adopted, and the survey method is questionnaire survey. In this survey, our group conducted an interview survey on the mental health problems of college students. During the investigation and interview, we made a specific division of labor for the investigators. Male students were responsible for 50 questionnaires and female students for 50 questionnaires, with a recovery rate of 98. After the questionnaire is collected, the number of the questionnaire is compiled. 1–49 questionnaires are for girls, and 50–98 questionnaires are for boys. The team leader shall hold a group analysis meeting and sort out and summarize the discussion results of the meeting. This paper will standardize and normalize the data. This paper introduces the data standardization and normalization in the process of data preprocessing from the specific meaning, differences, common methods and tools in practice. The feature is normalized by removing the mean and scaling to unit variance. To investigate whether there are differences between optimists and pessimists in optimism, life satisfaction, depression, coping style and health status. The independent sample t-test is carried out on the optimistic factors and pessimistic factors of optimists and pessimists in optimistic personality tendency, and the results are shown in Table 1.

**Table 1:** The difference test of optimism level between optimists and pessimists.

Factor	Group	M	SD	t	P
Optimistic factors	Optimist	11.03	0.97	20.07	0.000
	Pessimist	8.66	1.75		
Pessimistic factors	Optimist	1.74	1.12	−32.69	0.000
	Pessimist	6.82	1.66		

T-test results show that there are significant differences between optimists and pessimists in their optimistic factors and pessimistic factors. The scores of optimists in optimistic factors are significantly higher than those of pessimists, and those in pessimistic factors are significantly lower than those of pessimists.

Optimists score significantly higher than pessimists, while pessimists score significantly lower than pessimists. Thus, optimists are more optimistic than pessimists, and pessimists are more pessimistic than optimists. Researchers divide individuals into optimists and pessimists according to their positive or negative expectations for the future. Optimists usually think that good things will happen, while pessimists usually think that bad things will happen. The results of this study show that compared with pessimists, optimists are more likely to expect good things to happen in life and usually see the good side of things.

Optimism is not about the present or the past, but it is generally caused by speculation based on assumptions. Therefore, optimism is not only the result of cognitive judgment, but also the result of subjective desire. However, the result based on desire will actually affect people’s behavior now and in the future. A person’s natural genetic genes provide an optimistic baseline, and different people will have more or less differences in this respect; Optimists usually see the good, favorable or constructive side in real life (including the past, present and future), and this attitude can be reflected in practical actions and play a positive role, while pessimists are just the opposite. Such as life satisfaction, morality, emotion and self-esteem; And the measurement results are more likely to be optimistic than personality characteristics (Yadollahi et al., 2017).

Optimists have the courage to accept the reality and expect good things to happen in life, and usually see the good side of things, so they are more satisfied with life. Therefore, the higher an individual’s optimism level, the easier it is to have good expectations for the future, and the higher his satisfaction with real life, the less likely he is to have negative feelings of depression. Pessimists, on the other hand, are more willing to take the coping style of self-blame, fantasy and retreat. When faced with negative events, they often doubt their own abilities, and are prone to retreat and give up hope, thus falling into the abyss of depression.

### Factors influencing college students’ optimism

Stepwise regression method was used to test and analyze the predictive ability of these factors that affect college students’ optimism. Before the gradual regression, the apology was transformed into dummy variables, the scores of injury degree, optimism tendency and optimism were transformed into standard scores, and then the total score was calculated. With optimism as the dependent variable, the apology, optimism tendency and injury degree were sent into the regression equation, and the stepwise regression method was used for statistical analysis. The results are shown in Table 2.

**Table 2:** Stepwise multiple regression analysis of college students’ optimism.

Variable	R	$R^{2}$	ΔR	β
Degree of injury	0.508	0.134	0.036	−0.017^***^
Relationship (other)	0.427	0.134	0.045	−0.016^***^
Optimism tendency	0.458	0.106	0.044	−0.092^***^
Relationships (classmates)	0.417	0.265	0.037	0.1^***^
Apology	0.443	0.114	0.04	0.094^***^
Relationships (relatives)	0.441	0.164	0.037	−0.047^***^

*** *p < 0.001*.

There are six significant variables in the regression equation, the multiple correlation coefficient is 0.441, and the joint explanatory variance is 0.164, that is, the six variables in the table can jointly predict the 16.4% variance of college students’ optimism. As far as the interpretation of individual variables is concerned, the level of “injury degree” has the best predictive power.

Optimism tendency, relationship type, whether the aggressor apologizes or not, and the degree of harm are significant predictors of optimism. Specifically, the subjects who scored high on the optimism tendency scale also scored high on the optimism scale. “Relatives” and “friends” are more likely to get optimism. The higher the acceptance of apology, the more serious the injury and the lower the level of optimism. Optimism, as a kind of idiosyncratic optimism, is more influenced by some factors of the parties themselves, such as gender, age, etc.

This paper discusses whether gender, resilience and self-esteem can predict the emotional state of college students during the epidemic. The results of correlation analysis and multi-level regression analysis showed that the emotional status of college students had improved compared with the beginning of the epidemic, and the score of positive emotions was significantly higher than that of negative emotions. Self-esteem and psychological elasticity can significantly predict college students’ positive emotions. There are significant gender differences in the emotional state of college students, and the positive emotion of boys is significantly higher than that of girls.

Whether optimism or optimism tendency has a significant positive predictive power for happiness and life satisfaction, but a significant negative predictive power for depression. The results also show that compared with optimism and optimism tendency, optimism is superior to optimism tendency in predicting mental health. Good interpersonal relationship is conducive to enhancing college students’ sense of self-worth and strength, at the same time, it can reduce college students’ frustration, help them relieve their inner conflicts and anguish in time, vent their depression and pain, and reduce loneliness, loneliness, emptiness and fear. The benefits of optimism to oneself and others can guide students to choose whether to be optimistic or not, and help them master and apply the skills and techniques of others with optimism.

### The relationship between optimism and CSMH

#### Optimism and T-test analysis of mental health test results

T-test analysis of scores of various mental health test indicators of students with different levels of optimism is shown in Table 3.

**Table 3:** T-test analysis of various psychological test factors.

Variable	Good and above groups	Pass and the following groups	t	P
Suicide intention	4.179 ± 1.36	4.238 ± 2.55	0.638	0.1
Anxious	5.131 ± 2.25	4.694 ± 2.16	1.226	0.121
Depressed	4.251 ± 1.08	6.488 ± 2.33	0.598	0.141
Bigotry	4.49 ± 3.32	5.06 ± 1.96	−0.058	0.11
Inferiority	4.437 ± 1.15	4.687 ± 2.45	0.14	0.1
Sensitive	5.005 ± 2.25	7.109 ± 2.14	1.056	0.027
Social phobia	4.145 ± 2.18	4.294 ± 2.11	0.775	0.039
Rely on	5.409 ± 1.19	4.444 ± 1.98	0.852	0.106
Hostile attack	6.138 ± 3.36	6.682 ± 2.53	0.234	0.122
Somatization	6.071 ± 2.08	6.183 ± 2.47	1.094	0.017
Impulse	6.914 ± 1.96	5.103 ± 2.16	1.044	0.153
Force	4.894 ± 2.07	6.436 ± 2.87	0.373	0.038

From the above table, it can be seen that there are significant differences in four psychological factors, sensitivity, social fear, somatization and compulsion, between the above groups with good optimism test and the below groups with good optimism test, but there are no significant differences in anxiety. Most people who fail in physical fitness do not like sports, lack of physical exercise, resulting in lack of exercise, and often physical discomfort, limb weakness and other phenomena, which affect their normal life and study, and lead to bad emotions. All these indicate that mental health has a direct impact on physical health.

Among all the indicators of the students’ optimism test, the scores of strength scores of students without psychological distress are lower than those of students who may have serious psychological crisis, while the former is higher than the latter in all other test items. This suggests that if we want to keep a healthy body, we need to have a healthy mind. For college physical education workers, while we pay attention to physical education, we also need to try to integrate healthy and active psychological education courses to improve students’ mental health level, so as to promote the healthy development of students’ physical quality. We should formulate targeted exercise methods aimed at strengthening the overall physical quality, and help them improve their mental state through physical exercise; For students with general psychological problems, some exercises can be designed to enhance their physical flexibility, so as to regulate their mental health.

In the direct effect structure model, life stress has a significant inducing effect on psychological crisis. Optimism has a direct and significant intervention effect on psychological crisis. The results of this study also show that stress is an important cause of college students’ psychological crisis. The more serious the stress, the greater the impact on students’ mental health. It is proved that coping style plays an intermediary role, and it is also confirmed that optimism plays a role in stress events and psychological crisis through coping style, that is, it has a significant regulatory role.

#### Correlation analysis between optimism and mental health of college students

The correlation between optimism variables and mental health is analyzed, and the results are shown in Table 4 below.

**Table 4:** Correlation coefficient between optimism and mental health variables.

Variable	Emotional well-being	Psychological well-being	Social well-being	Positive function	Total amount table
Gender	0.175	−0.028	0.189^**^	−0.044^*^	0.012^*^
Grade	−0.117	0.01	−0.15^*^	0.357^*^	−0.027^*^
Specialized subject	−0.004	−0.137	−0.194^*^	0.032^*^	0.116^*^
Individual network scale	−0.012	−0.115	0.217	0.078	0.332
Individual net density	0.135	−0.269^**^	−0.125	0.19	0.313^**^
Point out degree	−0.166	−0.057	0.073	0.105^**^	0.138
Point-in degree	0.023^*^	−0.049	−0.15^*^	0.172^*^	0.275^*^
Approaching centrality	−0.13	−0.237^*^	−0.042^**^	0.091^**^	0.183^**^
Intermediary degree	0.14	−0.088	0.167	0.33	0.101

* *p < 0.05 and*

** *p < 0.01*.

As can be seen from Table 4, gender, grade and major are positively correlated with mental health scale, social well-being and positive function, while egocentric network density is negatively correlated with mental health continuum scale and mental well-being subscale. It can be seen that, except the scale of egocentric network, click-through degree and intermediary centrality, other optimistic variables are related to mental health. The optimism of college students affects their mental health level, but the correlation coefficient is small and the correlation degree is low.

From the analysis results, there is no significant demographic difference between emotional well-being and psychological well-being, but there are significant differences in the total score of mental health, social well-being and positive function. With the rise of grades, the psychological development of individual college students gradually matures and gradually shows their individual characteristics. One of the decisive factors of how much social capital an individual has is the size of the contact network that the actor can effectively use. Therefore, the larger the individual network, the more social capital, and the higher the level of mental health.

In the class network, students with high intermediary center are located on the geodesic line between two other actors. Therefore, the actor in the middle can control the interaction between these two actors who are not adjacent to each other, and has more interpersonal influence than other actors. Previous studies have shown that there is a positive correlation between egocentric network density and mental health, but there are differences in the relationship patterns. The positive predictive effect of high individual network density on mental health varies with different subjects. Interacting in a close network, people have a high degree of integration in the group, which may establish a sense of group identity, which is beneficial to individual mental health. Therefore, the students with low individual network density have high degree of network integration and strong sense of identity in the group, and their individual mental health level is high.

### Cluster analysis of CSMH

**Figure 4:**
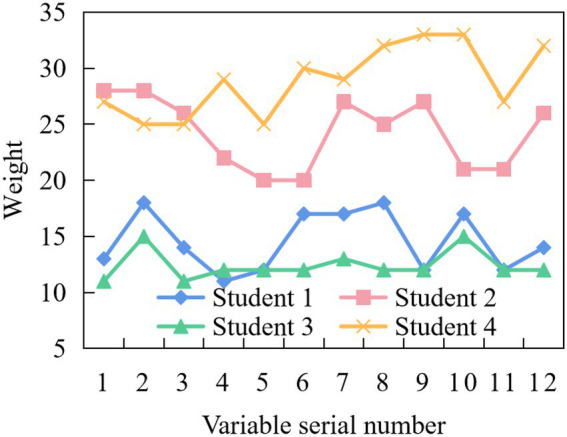
Student initial data.

In order to verify the application effect of the intelligent evaluation method of CSMH designed in this paper, four college students in a university were randomly selected as the application objects, and named as Applied Students 1 ~ 4, respectively. During the test process, the initial data of five application objects were collected according to the evaluation index system constructed in this paper, and the results are shown in Figure 4.

**Figure 5:**
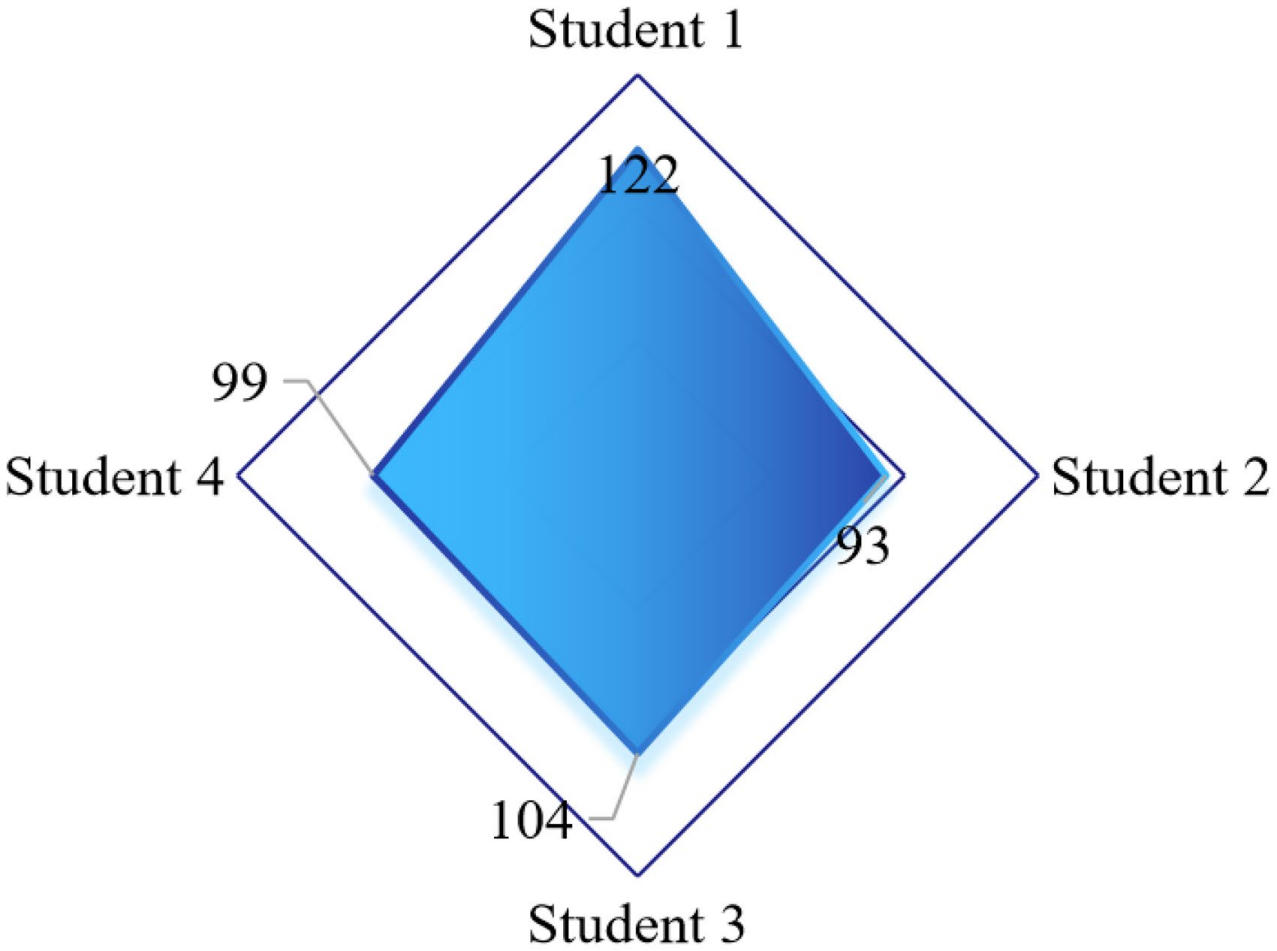
Calculation result of secondary comprehensive clustering coefficient.

Through the evaluation of CSMH, we can know the current level of CSMH and the main existing problems, and psychological counseling and related treatment according to the evaluation results can improve CSMH. Figure 5 shows that after the psychological state of each application object is treated according to the evaluation results of this paper, the mental health level of each application object shows a significant improvement, among which the mental health level of student 1 has the most significant improvement. Therefore, the application effect of this method is fully verified.

**Figure 6:**
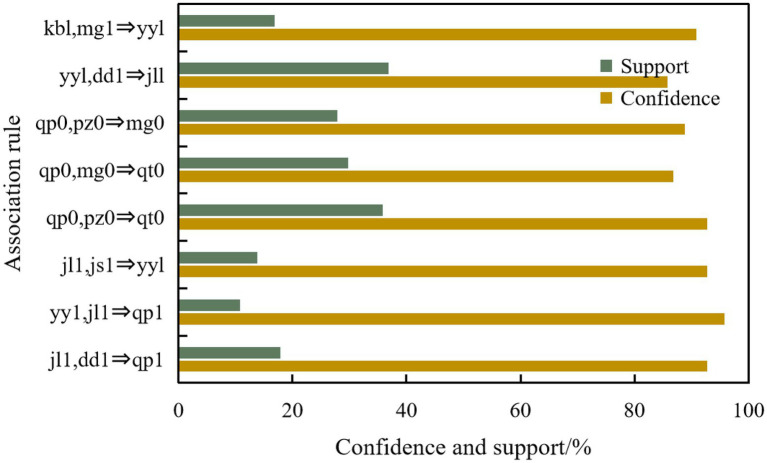
Partial association rules between psychological factors.

**Figure 7:**
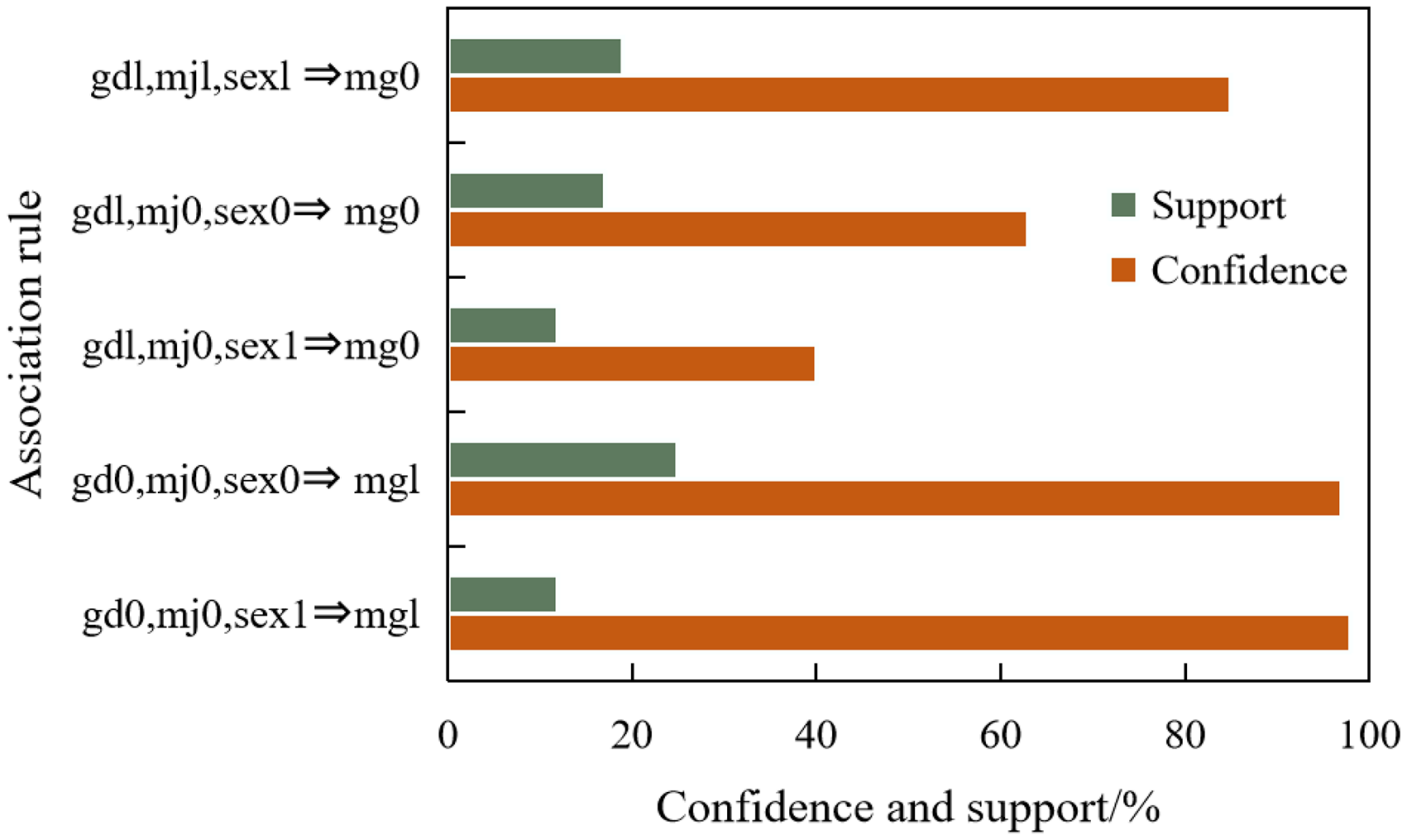
Partial association rules between interpersonal sensitive symptoms and basic information.

Combined with the above-mentioned ideas of college students’ psychological correlation analysis system, this paper makes correlation analysis on our mining objectives, finds out the potential relationship between those set factors, and then carries out mental health education for college students according to the results, and some association rules among sensitive symptoms of interpersonal relationships and basic information of students are shown in Figures 6, 7 respectively.

There is a high correlation between obsessive–compulsive disorder, anxiety, interpersonal sensitivity, paranoia and other symptoms; There is a high correlation between depression, sensitivity, terror, psychosis and other symptoms.

Data selection is based on a clear task direction and a certain understanding of the data itself. Find out the dominant characteristics of the data itself to reduce the overall scale of the data, so as to streamline the data to the greatest extent while maintaining a certain data integrity. In the mental health system, in view of various problems existing in the data set (such as standardization, ambiguity, repeatability, etc.), pre-processing the student data such as noise reduction, missing value filling, and duplication removal can further strengthen the stability of the system in the following mining analysis, and the purpose of optimizing the system has been achieved. In the mental health system, the ability level in this aspect is expressed by the ability value.

**Figure 8:**
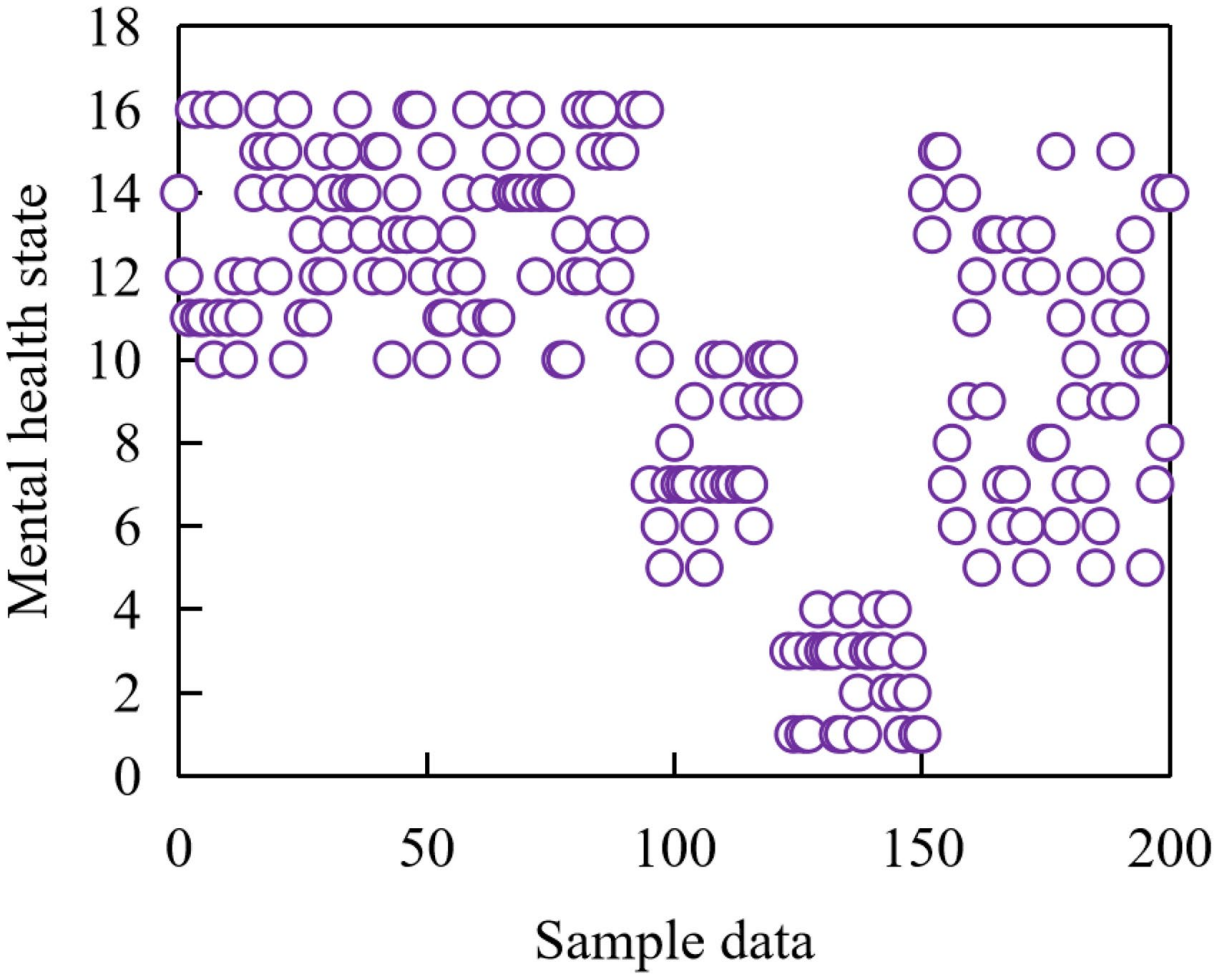
Cluster analysis chart of students’ mental health status.

In this example, the best number is 3 classes, and the cluster distribution table shows the frequency of each class. The data shows that there are 186 students in cluster 1, 166 students in cluster 2, and the remaining 64 students in cluster 3, among which 13 students are excluded from the system due to lack of data. Meanwhile, a two-dimensional distribution map of cluster analysis is obtained from the data, as shown in Figure 8.

At the same time, under the credit system and flexible academic system, students’ class functions are weakened, the concepts of departments, grades and majors are weakened, the curriculum arrangement is relaxed, students’ non-intellectual factors are changed, and students’ independent and free space is increased. Therefore, students’ learning is bound to need correct guidance and guidance. With the trend that students’ socialization, entertainment, study and social work are gradually leaving the campus and going to the society, the limitations of the traditional mode of focusing on the inside of the school and the system itself will become more and more prominent.

Actively create conditions, overcome difficulties, and maximize the participation of more students in various activities of colleges and universities, so as to meet the needs of students as much as possible, achieve educational goals, cultivate college students’ healthy psychology, and promote their healthy growth.

Given a set of multidimensional data points, the data points are usually not evenly distributed in the data space. The algorithm distinguishes sparse and dense areas in space to discover the global distribution pattern of data sets. If the data points in a cell exceed a certain input model parameter, the cell is dense. This model may be the density distribution function of data points in space or others, or it may automatically determine the number of clusters based on standard statistics.

**Figure 9:**
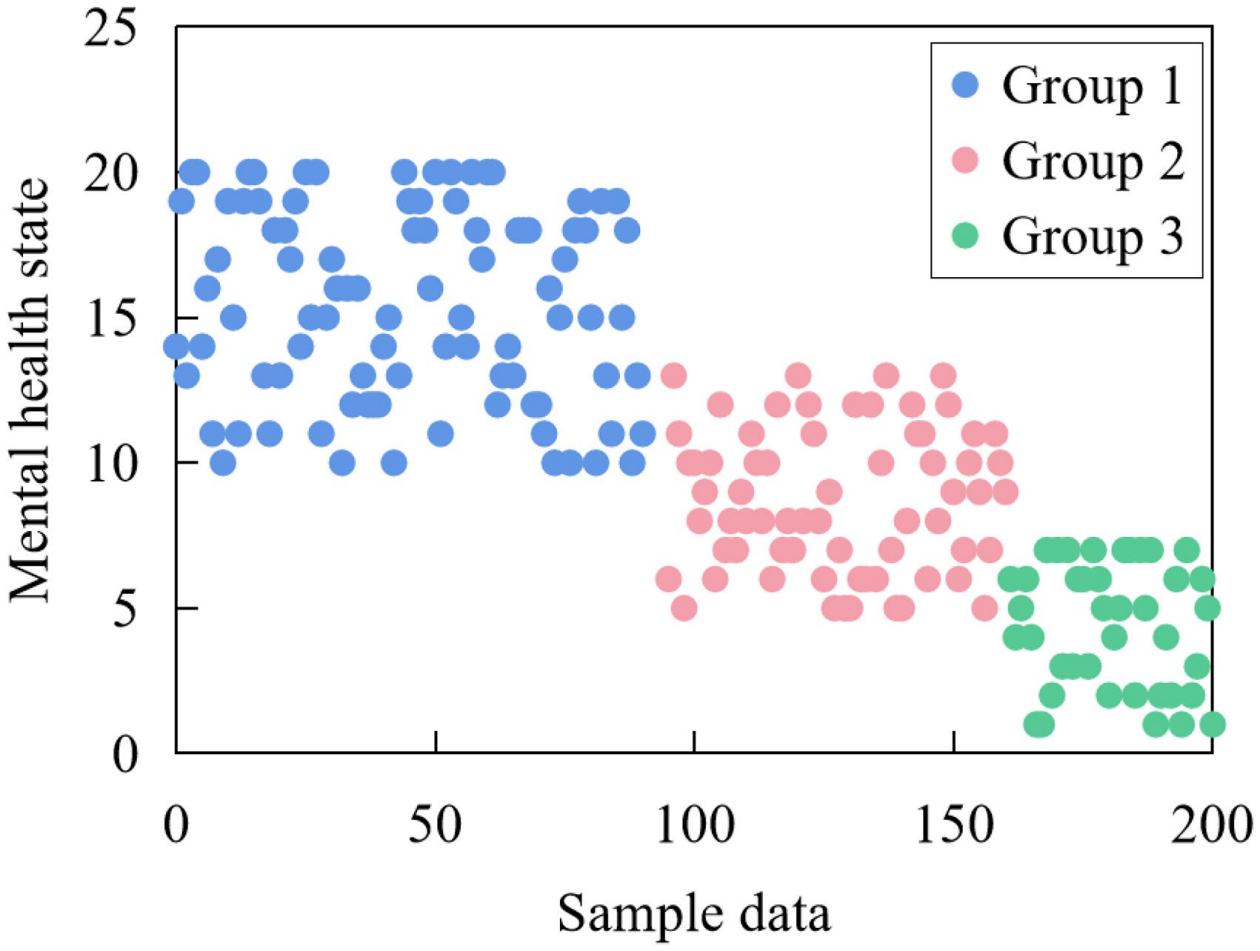
Results of cluster analysis.

The new object can be assigned to the cluster with the most similar specimen, and the attribute of the object assigned to a cluster can be predicted according to the attribute of the specimen in that cluster. The final result of cluster analysis is shown in Figure 9.

According to the cluster proportion diagram of each attribute, it is easy to make students have a greater psychological impact on their adaptability to the environment, while compared with the previous attributes, it is suggested that students’ family background and students’ actual situation should be taken into account when conducting mental health education.

In the seemingly indistinguishable teaching environment, the learning effect varies from person to person and changes with time. This reflects that learning performance is not only driven by teaching environment, teaching methods and students’ inherent ability, but also related to students’ dynamic behavior to a great extent. Applying students’ individual behavior mode to effectively predict students’ learning performance can provide timely supervision and feedback, provide personalized service for students and optimize teaching effect. While retaining the uniqueness of a single task, it can learn from multiple tasks at the same time by capturing the relationship between tasks, which can improve the generalization performance and interpretability of the model while improving the prediction effect of a single task and the whole task.

## Research suggestions

Through the above analysis and discussion, we can see that the causes of college students’ psychological problems are complicated, and the solution of the problems should be considered in the context of the whole society. Positive and optimistic attitude will produce optimistic and upward mood and passionate behavior. A positive and optimistic attitude helps to successfully deal with daily affairs and can make life more optimistic. And minimize worries and negative thoughts. Positive and optimistic attitude can make people see the good aspects of life and become optimistic and aggressive.

We should strengthen psychological quality education. College students are faced with a series of adaptation problems from middle school to university, but they mainly adapt to changes. Including the change of one’s own role, interpersonal relationship and study and living environment. Empathy helps the case to re-evaluate the injury incident, so as to know that there may be some reasons for the aggressor’s aggression, and it can also make the case know that the aggressor may also be in self-blame and guilt. While emphasizing justice and care, school moral education should further emphasize the importance of optimistic psychological education, appropriately increase the content of optimistic psychological education, and focus on cultivating individual optimistic psychological quality, which is very necessary for the physical and mental health of teenagers.

The intervention effect of optimism on stress events and psychological crisis is of great practical significance to the current stress management and psychological crisis intervention of college students. Optimism can promote individual psychological and physiological health, increase people’s psychological capital, feel more social support, and make people believe that the results will be better. For college students, optimism is of great practical significance in forming a healthy physiological and psychological state, enhancing their strength and virtue in human nature, and helping them to develop themselves continuously. Do not make excuses for your failure, do not be depressed by failure, feel the joy of struggle in adversity, overcome all kinds of doubts about yourself, life and society, develop a healthy cultural mentality of being positive and enterprising, and establish an optimistic attitude towards life.

A good campus cultural environment has a distinct educational function, especially the cultivation and guiding function of college students’ personality, which cannot be replaced by other educational forms. Campus cultural environment includes two meanings: one is static cultural environment, the other is dynamic cultural environment. Colleges and universities should make a unified layout and overall planning. Under the guidance of aesthetics, psychology, pedagogy and other related principles, the campus should be carefully designed and reconstructed to keep the campus beautiful, elegant and quiet, and strive to make every corner of the campus filled with the breath of mental health education. Form a mental health education system that closely combines in-class and out-of-class, education and guidance, consultation and self-help. At the same time, the choice of course content and teaching methods must be realistic and close to students’ reality. Therefore, mental health education is a necessary course to help contemporary college students know and evaluate themselves with an objective, comprehensive and dichotomous viewpoint, constantly improve themselves in study and practice, and form and develop their excellent personality.

According to the analysis of the causes of college students’ psychological problems, we can know that the occurrence of students’ psychological problems is closely related to the students’ family economic situation, the employment market situation and the education situation of schools at all levels. These aspects are closely related to the systems, policies and supervision made by the government. As a teacher or consultant engaged in mental health work, they must have graduated from university with professional qualification certificate, have good experience in teaching or consulting, and master the relevant knowledge and skills of mental health education or consulting comprehensively and systematically. At the same time, governments at all levels should expand the proportion of education in the financial budget, raise venture capital from various aspects, and give greater support to outstanding college students’ self-employment; On the basis of ensuring the overall fairness of the society, relieve the psychological pressure of college students’ employment. In addition, the government should strengthen the social and cultural construction, crack down on cybercrimes, purify the social and cultural environment, truly fulfill its duties, and provide a social environment conducive to the healthy development of college students’ body and mind.

Social relations can strengthen identity and identity, and improve the degree of social integration. With a high degree of concentration and strong cohesion, the network can convince and realize that the actor is a valuable individual and a member of a social group sharing similar interests and resources. Establish positive interpersonal relationships, expand the scale of optimism, perceive wider social support, and improve positive emotional experience and life satisfaction in life. Create a good class environment and atmosphere. In daily life and study, if students encounter problems or difficulties, the close network relationship between classes can provide students with stronger emotional support and obtain social resources in the network. To promote class unity, students can also be encouraged to get out of class, participate in social activities organized by schools, colleges and other organizations, join societies, expand the scale of individual networks, build social networks at the school level, and improve the level of network integration.

Taking part in more sports helps to improve mental state. With the development of the national fitness campaign, more and more people are involved in sports. What kind of fitness method is the most suitable? In addition to considering age, occupation, living environment and other factors, taking personal personality factors into account will achieve twice the result with half the effort. Sports psychology research shows that different items have different effects on psychology. In real life, some people lack the psychological adjustment and adaptability that normal people have, or show obvious personality defects and emotional defects. Through targeted and appropriate exercise, they can correct bad personality defects and improve their psychological and mental state.

## Conclusion

There are significant differences between optimists and pessimists in their optimistic factors and pessimistic factors. Optimism is a scientific working method. Optimists prefer a “maximization” decision-making method, also known as the optimism criterion. When facing the decision-making problem with unknown situation, never give up any opportunity to obtain the best result, and choose the decision-making scheme with the optimistic attitude of striving for the best of the best. The scores of optimists in optimistic factors are significantly higher than those of pessimists, and those in pessimistic factors are significantly lower than those of pessimists. There are six significant variables in the regression equation, the multiple correlation coefficient is 0.441, and the joint explanatory variance is 0.164, that is, the six variables in the table can jointly predict the 16.4% variance of college students’ optimism. Stress is an important cause of college students’ psychological crisis. The more serious the stress, the greater the impact on students’ mental health. Gender, grade and major are positively correlated with mental health scale, social well-being and positive function, while egocentric network density is negatively correlated with mental health continuum scale and mental well-being subscale. However, the research has certain limitations. The generalization performance and interpretability of the model have not been effectively verified. At the same time, the prediction effect of single task and the whole task is not supplemented. Therefore, further modifications are needed in the future research and analysis.

## Data availability statement

The raw data supporting the conclusions of this article will be made available by the authors, without undue reservation.

## Author contributions

HZ and WN put forward the idea and design together and did the preliminary draft together. HZ did the method design. WN did the data analysis and data sorting. All authors contributed to the article and approved the submitted version.

## Funding

The work was supported by Qiqihar University. (1) Humanities and Social Sciences Research Project of the Ministry of Education 21JDSZ3118. (2) Humanities and Social Sciences Research Project 20YJA880089 of the Ministry of Education. (3) Educational Science Research Project ZD201810 of Qiqihar University.

## Conflict of interest

The authors declare that the research was conducted in the absence of any commercial or financial relationships that could be construed as a potential conflict of interest.

## Publisher’s note

All claims expressed in this article are solely those of the authors and do not necessarily represent those of their affiliated organizations, or those of the publisher, the editors and the reviewers. Any product that may be evaluated in this article, or claim that may be made by its manufacturer, is not guaranteed or endorsed by the publisher.

## References

[ref1] Debate D. G. Gatto A. Rafal G. (2018). The effects of stigma on determinants of mental health help-seeking behaviors among male college students: an application of the information-motivation-behavioral skills model. Am. J. Mens Health 12, 1286–1296. doi: 10.1177/1557988318773656, PMID: 29749301 PMC6142134

[ref2] Fruehwirth J. C. Biswas S. Perreira K. M. (2021). The covid-19 pandemic and mental health of first-year college students: examining the effect of covid-19 stressors using longitudinal data. PLoS One 16:e0247999. doi: 10.1371/journal.pone.0247999, PMID: 33667243 PMC7935268

[ref3] Fu W. Yan S. Zong Q. Dan A. L. Lv C. (2021). Mental health of college students during the covid-19 epidemic in China. J. Affect. Disord. 280, 7–10. doi: 10.1016/j.jad.2020.11.032, PMID: 33197782 PMC7656159

[ref4] Góngora A. S. Isabel T. D. Sofiane H. Miguel L. C. Calvo B. D. Morón N. L. (2018). Data mining algorithms and techniques in mental health: a systematic review. J. Med. Syst. 42:161. doi: 10.1007/s10916-018-1018-2 30030644

[ref5] Jorge B. Susana M. Orietta E. Arnol G. Jaime O. Takeshi A. (2016). Suicide detection in Chile: proposing a predictive model for suicide risk in a clinical sample of patients with mood disorders. Rev. Bras. Psiquiatr. 39:1. 27783715 10.1590/1516-4446-2015-1877 PMC7112738

[ref6] Qi S. Hua F. Xu S. Zhou Z. Liu F. (2021). Trends of global health literacy research (1995–2020): analysis of mapping knowledge domains based on citation data mining. PLoS One 16:0254988. doi: 10.1371/journal.pone.0254988 PMC8351965 34370749

[ref7] Shervin A. Maryam M. L. (2018). Violence exposure and mental health of college students in the United States. Behav. Sci. 8:53. doi: 10.3390/bs8060053 PMC6027217 29882926

[ref8] Tang J. Xu H. (2016). Analysis of self-management education on improving the symptoms of female college students with primary dysmenorrhea in Shaoyang. J. Central South Univer. (Med. Sci.) 41:434. doi: 10.11817/j.issn.1672-7347.2016.04.016 27241157

[ref9] Tang Q. Zhao Y. Wei Y. Jiang L. (2021). Research on the mental health of college students based on fuzzy clustering algorithm. Security Commun. Networks 2021, 1–8. doi: 10.1155/2021/3960559

[ref10] Taylor D. E. (2019). College students and nature: differing thoughts of fear, danger, disconnection, and loathing. Environ. Manag. 64, 79–96. doi: 10.1007/s00267-019-01172-9, PMID: 31076829 PMC6598941

[ref11] Vaughn A. A. Drake R. R. Haydock S. (2016). College student mental health and quality of workplace relationships. J. Am. Coll. Heal. 64, 26–37. doi: 10.1080/07448481.2015.1064126, PMID: 26151646

[ref12] Wang X. (2020). Research on mental health education for college students. Intern. J. Soc. Sci. Educ. Res. 3, 153–157.

[ref13] Wang Y. H. Shi Z. T. (2018). The influence of social support on sexual mental health of female college students. Medicine 97:e11525. doi: 10.1097/MD.0000000000011525, PMID: 29995823 PMC6076082

[ref14] Wei M. Liu Y. Liang F. Huang J. Xia R. (2020). The application status of horticultural therapy in the study of college students' physical and mental health. J. Landscape Res. 12, 108–110.

[ref15] Wongkoblap A. Vadillo M. A. Curcin V. (2017). Researching mental health disorders in the era of social media: systematic review. J. Med. Internet Res. 19:e224. doi: 10.2196/jmir.7215 28663166 PMC5509952

[ref16] Xiao T. Jiao C. Yao J. Yang L. Zhang J. (2021). Effects of basketball and baduanjin exercise interventions on problematic smartphone use and mental health among college students: a randomized controlled trial. Evid. Based Complement. Alternat. Med. 2021, 1–12. doi: 10.1155/2021/8880716 PMC7864751 33574886

[ref17] Xue B. Liu T. (2018). Physical health data mining of college students based on drf algorithm. Wirel. Pers. Commun. 102, 3791–3801. doi: 10.1007/s11277-018-5410-5

[ref18] Yadollahi A. Shahraki A. G. Zaiane O. R. (2017). Current state of text sentiment analysis from opinion to emotion mining. ACM Comput. Surv. 50, 1–33. doi: 10.1145/3057270

[ref19] Ying G. (2021). Methods of mental health education for chinese college students in the new era. Educ. res. front. 11:4.

[ref20] Zhang Q. Chen F. (2019). Current situation of college students’ “staying at home” and their mental health level. guide sci. educ. 2, 165–168.

